# Machine Learning for Predicting Mortality in Transcatheter Aortic Valve Implantation: An Inter-Center Cross Validation Study

**DOI:** 10.3390/jcdd8060065

**Published:** 2021-06-04

**Authors:** Marco Mamprin, Ricardo R. Lopes, Jo M. Zelis, Pim A. L. Tonino, Martijn S. van Mourik, Marije M. Vis, Svitlana Zinger, Bas A. J. M. de Mol, Peter H. N. de With

**Affiliations:** 1 Department of Electrical Engineering, Eindhoven University of Technology, 5612 AE Eindhoven, The Netherlands; s.zinger@tue.nl (S.Z.); p.h.n.de.with@tue.nl (P.H.N.d.W.); 2 Department of Biomedical Engineering and Physics, Amsterdam UMC, University of Amsterdam, 1105 AZ Amsterdam, The Netherlands; r.riccilopes@amsterdamumc.nl; 3 Department of Radiology and Nuclear Medicine, Amsterdam UMC, University of Amsterdam, 1105 AZ Amsterdam, The Netherlands; 4 Department of Cardiology, Catharina Hospital, 5623 EJ Eindhoven, The Netherlands; jo.zelis@catharinaziekenhuis.nl (J.M.Z.); pim.tonino@catharinaziekenhuis.nl (P.A.L.T.); 5 Heart Centre, Amsterdam UMC, University of Amsterdam, 1105 AZ Amsterdam, The Netherlands; m.s.vanmourik@amsterdamumc.nl (M.S.v.M.); m.m.vis@amsterdamumc.nl (M.M.V.); b.a.demol@amsterdamumc.nl (B.A.J.M.d.M.)

**Keywords:** aortic valve disease, machine learning, inter-center cross-validation, one-year mortality prediction, outcome prediction, prognosis, TAVI, transcatheter aortic valve implantation

## Abstract

Current prognostic risk scores for transcatheter aortic valve implantation (TAVI) do not benefit yet from modern machine learning techniques, which can improve risk stratification of one-year mortality of patients before TAVI. Despite the advancement of machine learning in healthcare, data sharing regulations are very strict and typically prevent exchanging patient data, without the involvement of ethical committees. A very robust validation approach, including 1300 and 631 patients per center, was performed to validate a machine learning model of one center at the other external center with their data, in a mutual fashion. This was achieved without any data exchange but solely by exchanging the models and the data processing pipelines. A dedicated exchange protocol was designed to evaluate and quantify the model’s robustness on the data of the external center. Models developed with the larger dataset offered similar or higher prediction accuracy on the external validation. Logistic regression, random forest and CatBoost lead to areas under curve of the ROC of 0.65, 0.67 and 0.65 for the internal validation and of 0.62, 0.66, 0.68 for the external validation, respectively. We propose a scalable exchange protocol which can be further extended on other TAVI centers, but more generally to any other clinical scenario, that could benefit from this validation approach.

## 1. Introduction

Aortic valve stenosis (AS) is one of the most common valve diseases in the developed world, primarily impacting the elderly population [1]. When symptomatic, the disease has a devastating impact when left untreated, rapidly increasing the risk for heart failure, which can lead to death in many cases. The most common cause of AS is calcification, resulting from a network of pathological processes such as endothelial dysfunction and injury, inflammation, synthetic and osteogenic dedifferentiation, pro-fibrotic response of valvular interstitial cells and degradation of the extracellular matrix by proteolytic enzymes. The calcium deposits on the valve leaflets result in impaired leaflet motion and subsequent obstruction and/or regurgitation of blood flow. Another common cause is cardiac fibrosis which leads to heart valve thickening which decisively contributes to heart valve failure in addition to calcification. As a consequence, the left ventricle tries to compensate for the reduction in cardiac output by hypertrophy. Progressive narrowing of the valve will result in symptoms while conducting daily activities, like angina, dizziness and dyspnoea.

**Figure 1:**
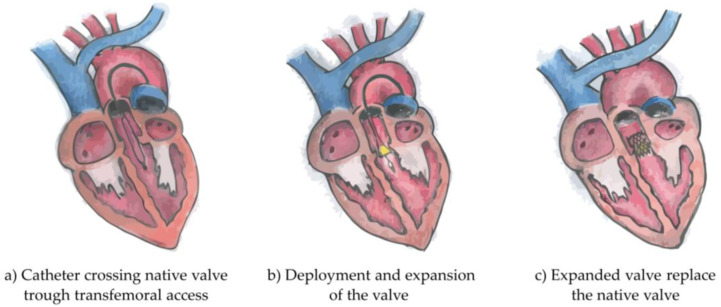
Illustration of the three phases of Transcatheter Aortic Valve Implantation intervention. Originally the catheter crosses the native valve trough transfemoral access (**a**), follows the deployment and expansion of the valve (**b**) and finally the expanded valve replaces the native valve (**c**).

Traditional treatment of severe AS consists of surgical aortic valve replacement (SAVR). However, in the past decades, a less invasive and more innovative approach has been developed and approved, initially for use only in high surgical-risk AS, and later extended to mild- and moderate-risk AS (risks calculated according to the established surgical risk scores). This approach is a minimally invasive heart procedure which requires the implantation of an artificial valve, as shown in Figure 1. The procedure is known as transcatheter aortic valve implantation (TAVI) or transcatheter aortic valve replacement (TAVR), and has currently become a routine treatment worldwide.

Strict patient selection is performed by a multi-disciplinary team to select patients best suited for TAVI. The selection is achieved by exploiting both current imaging technologies and recent clinically related research, which are jointly discussed in an inter-disciplinary meeting with medical experts and by using modern planning and treatment support tools [2,3,4].

The global objective of the research in this paper is to improve on the prediction of possible benefits for TAVI patients (further information is available in the Supplementary Materials), such that unnecessary treatment and reduction of the quality of life is avoided for patients not benefiting from TAVI, while giving more certainty to the benefiting patients by the confirmation of the prediction of improvements. A second reason for this research is found in the use of the mortality prediction. If the prediction score is high, the medical team of experts can discuss alternative treatments or medications and maintain the quality of life and life expectancy as high as possible. Alternatively, if the score is low, the medical team can use this as a confirmation to further proceed with TAVI and stimulate the patients for further motivation of treatment. Whereas the previous motivations are mostly related to the patient, the proposed study also provides benefits to the medical centers by facilitating the patient selection, reducing waiting lists, optimizing the use of the resources and improving the clinical workflow [1].

Research on mortality prediction and risk models has been addressed in literature. However, current risk models have only limited accuracy in predicting TAVI outcomes [5]. Several models originally intended for SAVR, thus not specifically intended for TAVI, have been developed in recent decades and are currently used for risk assessment. It is noteworthy and important to mention EuroSCORE, EuroSCORE II and the STS (Society of Thoracic Surgery) scores [6,7,8,9], since they are in widespread use worldwide. More specific TAVI predictors are also available, of which a relevant example is the TAVI-specific TVT registry score [10,11,12]. Considering that these are procedural, in-hospital or 30-day mortality predictors, the time interval is not clinically relevant to establish whether the TAVI procedure is suited for long-term patient survival. In fact, one-year mortality has been identified by the medical experts as the life expectancy threshold, above which the TAVI procedure is appropriate to be performed. However, one-year mortality for patients treated with TAVI is even more challenging [13] and current predictors [14,15] have not been extensively validated across multiple centers, reaching a validation sufficient to be used for the clinical decision-making. Furthermore, external validations are therefore needed to assess the predicting potential of one-year mortality for TAVI and this is a challenging task, as will become evident from this study.

The previously mentioned clinical risk scores and predictors for one-year mortality rely on conventional statistical regression approaches [16]. However, in recent years, more advanced machine learning techniques (ML) have shown competitive predictive value [17]. Especially when data complexity arises and a large amount of data are involved, ML techniques can outperform conventional regression models [18]. Recently, two ML methods have been developed and published, exploiting gradient boosting on a decision tree algorithm (GBDT) [19,20], to predict one-year mortality for patients treated with TAVI. The GBDT techniques were validated on retrospective patient data from single centers. However, external validations were not performed. The research we performed in this manuscript is meant as a natural consequence and continuation of these single-center researches previously mentioned. This cross-validation study was facilitated by the ongoing collaboration between the two medical and technical universities.

External validations are necessary to assess the generalization capabilities of a prediction model on other similar populations, albeit belonging to different centers. Most often this validation process requires the involvement of specific ethical committee protocols to permit the exchange of the patient data specifically for validation purposes. This involves long administrative procedures that are difficult to implement, especially in those scenarios where data sharing policies are intrinsically limited (e.g., General Data Protection Regulation).

**Figure 2:**
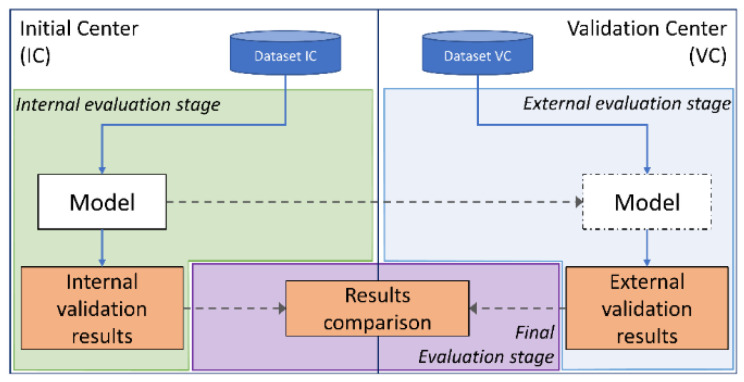
Diagram of the inter-center cross-validation. The entire exchange process is obtained by organizing a cross-validation protocol in three main stages, which requires the exchange of the models and their evaluation. Each model is validated on the initial center and on the validation center. Finally, a comparison between the internal validation and the external validation results is performed to evaluate the performance of each model on an unseen external center dataset.

An alternative solution to the data sharing limitations is exchanging the model and recreating the same data processing pipeline to perform the model validation, without any need of data sharing, as shown in Figure 2. In this research, a model-exchange approach has been adopted to avoid the exchange of any patient data, which would have otherwise delayed and hindered the validation process. The value of such a validation approach is considerable both from a perspective of robustness and for thoroughly testing the models.

To the best of our knowledge, this is the first extended cross-validation for predicting TAVI outcomes, performed across centers while not involving any data exchange. Some studies have been performed on national registries containing multiple-center data [21,22,23], however, data exchange was required and cross-validations to evaluate each model robustness across multiple centers were not performed. Summarizing, the clinical and technical relevance of what is presented in this study is remarkably important for the TAVI procedure and for further studies that will require external validations across multiple centers with insurmountable data privacy restrictions. This study is not meant to be a comparison between the models of both centers, since clinical features, data processing and classifiers optimization were commonly adopted to guarantee the highest similarity across the center’s experiments. In fact, the performance of various models which are later validated on data of other centers can also be highly influenced by population differences and different data distributions. This study aims also to analyze this problem, for dedicated models for the TAVI procedure, by performing several experiments with different machine learning classifiers, in order to quantify their robustness and identify the classifier with the lowest performance drop caused by data of another center.

This work has multiple clinical and technical contributions.

- First, we create multiple different mortality prediction models for the TAVI procedure per center, based on state-of-the-art machine learning techniques.
- Second, we optimize the TAVI models to better perform on the validation set and we evaluate their predicting potential on the test set of each original center.
- Third, we cross-validate all TAVI models, involving the two centers with different populations and we compare their predicting capability with respect to their internal evaluation.
- Ultimately, this study proposes an exchange protocol which can be extended to be used in further clinical use cases that can benefit from external validations or cross-validations.

## 2. Materials and Methods

In this section, details about the dataset, the inter-center cross-validation protocol, and the experimental setup are provided.

### 2.1. Datasets

The dataset available at the Eindhoven University of Technology (Technische Universiteit Eindhoven, TU/e) was obtained from the Catharina Hospital of Eindhoven (Catharina Ziekenhuis Eindhoven, CZE) and consisted of 631 consecutive TAVI procedures that were performed between January 2015 and December 2018. From this point onwards, the collaboration, based on the Eindhoven MedTech Innovation Center (e/MTIC) consortium, between TU/e and CZE is referred to in this work as CZE-TU/e. The dataset available at the Amsterdam UMC-Location AMC (Amsterdam University Medical Center, Location AMC) consisted of 1300 TAVI procedures performed between October 2007 and April 2018. Most procedures were performed by using the following models of artificial aortic valves:

- SAPIEN XT and SAPIEN 3, Edwards LifeSciences (Irvine, CA, USA);
- CoreValve and CoreValve Evolut, Medtronic (Minneapolis, MN, USA).

Transfemoral access was the most widely used access site to perform the procedures. However, a portion of the procedures, for both CZE and AMC, were performed by using an alternative access site. As later discussed, valve type and manufacturer and the access site have not been included as clinical features for this analysis study, since they are not considered patient data but procedure-related data, which are more important in procedural mortality than for one-year mortality. In recent years, procedural mortality significantly dropped and, in this study, the procedural time intervals of the two centers is substantially different (2007–2018 against 2015–2018). Furthermore, clinical protocols for patient selection and for the procedure also changed over time and across different centers. For these reasons, we have chosen to leave out any procedural data to obtain a fairer comparison [24]. For instance, the artificial aortic valve is itself also subject to changes due to the research which leads to technical advancements, whereas physiological information is less prone to changes across time and across multiple centers.

Prior to performing any analysis, datasets of both centers were divided into two different groups, representing two different categories of the population. For each group, an identification class (or label) was assigned accordingly to the patient survival after one year from the date of the TAVI procedure. The two main groups were identified as:

- Survived at one year (1171 patients for AMC and 564 patients for CZE-TU/e),
- Non-survived at one year (129 patients for AMC and 67 patients for CZE-TU/e).

The mortality information was collected after one year from the procedure through the census of the national population of the Netherlands for CZE-TU/e and during a follow-up study for AMC. Time-to-event has a median value of 55 days (IQR = 153 days) for AMC and of 93 days (IQR = 176 days) for CZE-TU/e.

The reporting of this paper adheres to the “transparent reporting of a multivariable prediction model for individual prognosis or diagnosis” (TRIPOD) guidelines [25]. The TRIPOD statement can be found in the Supplementary Materials.

### 2.2. Clinical Data Processing

**Figure 3:**
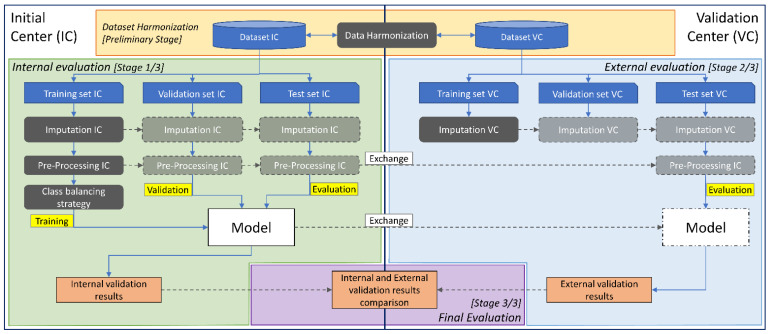
Detailed diagram of the inter-center cross-validation. Training, validation and evaluation are shown in yellow color, while data harmonization, imputation, pre-processing and class-balancing strategy steps are shown in grey color. The inter-center cross-validation protocol is organized in three stages: internal evaluation, external evaluation and final evaluation. The first two stages share a common clinical data processing, a common validation order and evaluation metrics. The internal evaluation stage (**left**) and the external evaluation stage (**right**) share a common processing chain including imputation and pre-processing steps. The final evaluation stage (**bottom**) is the last stage which is necessary to compare the internal validation results with the external validation results. At the first two stages, each model is evaluated, while at the final stage, both internal and external validation results are compared for a final performance assessment.

Clinical data are harmonized, imputed, pre-processed and in some cases oversampled before being used to train the classifiers. In this section all the steps are discussed with reference to Figure 3.

#### 2.2.1. Data Harmonization

The data-harmonization stage is a preliminary yet important and extensive step that implements the alignment of the dataset to a common representation. In fact, only patient data that was common (cross-available) to both centers was included in the study. When the clinical data was represented in a different form, the harmonization step was required to match the different data representations to realize a common one. Clinical data are both represented as numerical and as categorical information. Whereas numerical data can be represented in different units easily convertible and interchangeable, categorical data can be expressed with a different number of instances. In case the number of categories were different, we reduced the number of categories to an amount common to both datasets. The main harmonization steps that were performed at this preliminary stage are explained in detail in Table A2 (Appendix B). As a natural consequence, each dataset during its collection process is afflicted with a certain number of missing values. In this research, we initially discarded all features that had 80% or more missing values, for at least one of the two centers. Missing values for all the features that were included in the study (therefore with a percentage of missing values lower than 80%) were imputed, as explained in the next section. This threshold was jointly chosen by the two centers to guarantee that an adequate number of important features were available, despite the amount of missing information. The main reasons why data was missing is because of random events leading to the data not being collected, but also because of non-random events [26,27]. For this last scenario, a dependency between the data collection and clinical protocols can exist, which can affect the collection of that type of data, not considering it of crucial importance for the decision making of a specific patient. This type of missing information can be very center dependent since clinical hospitals and academic medical centers often do not share the same clinical protocols.

**Figure A2:**
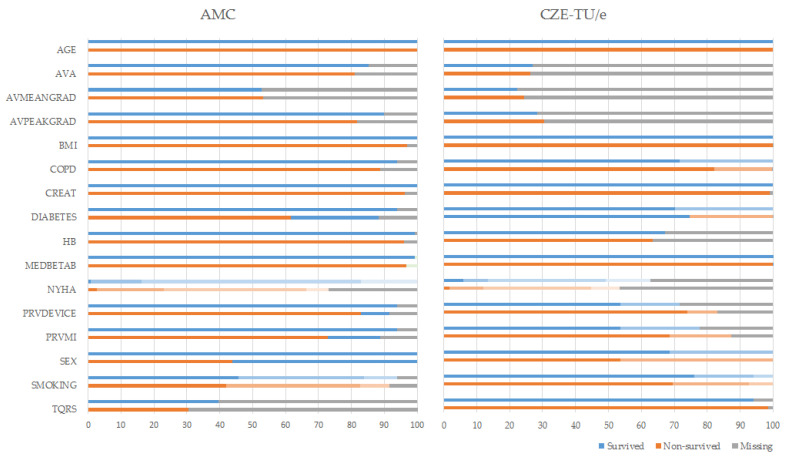
Normalized bar plots with distributions of the categorical features of the two different centers. Non-survived and survived patient distributions are shown in orange and blue, respectively. For all features, missing values are reported in grey, numerical values are identified with pure orange and pure blue, while categorical instances are represented with graduated levels of orange and blue.

A total of 16 clinical features were used in the study and are all listed in alphabetical order in Table A3 (Appendix B). Further information about quantity of missing values are shown in Figure A2 (Appendix B).

#### 2.2.2. Data Imputation

**Figure 4:**
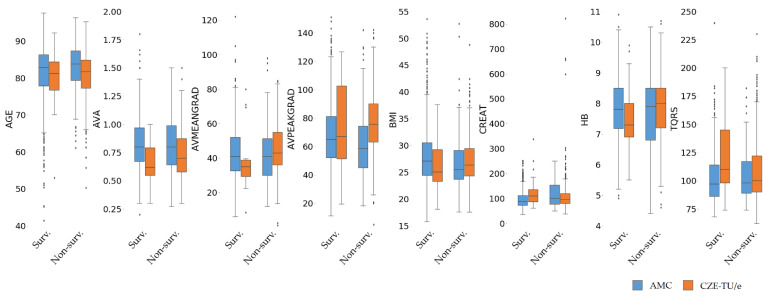
Boxplot distributions of the numerical features for the two different centers. AMC and CZE-TU/e feature distributions are shown in blue and orange, respectively. Imputed data for missing values are not included to avoid influence on the original data distribution.

Missing values were then imputed with the mean value for numerical features and with the mode of the instances for categorical features. The survived and non-survived classes were not considered for the computation, leading to the consideration of a unique mean or mode per feature and per center. These unique values per feature and per center were then used to impute the training, validation and test set of each respective center. As known, taking the median value is robust to outliers. However, as observed in Figure 4, data in the survived and non-survived distributions have shown that the median value does not incorporate a large number of outliers, which represent a large sub-population of both two centers. This is the reason replacing numerical missing values with a mean value was preferred, since the mean includes the outliers and has shown a higher consistency in its value, with respect to the survived and non-survived distributions across the two centers. This approach was also chosen because of its low computational complexity, but also because it is a well-known standard approach in the machine learning field. Furthermore, we considered using imputation values taken from the original center data (center-specific imputation), since slightly different data distributions over the two centers did emerge. As specified in the previous section, different clinical protocols are adopted across the two centers, and also, for this reason, a center-dependent approach was preferred. Further information about data distributions is listed in Table A3 (Appendix B) and shown in Figure 4.

#### 2.2.3. Data Pre-Processing

Pre-processing for most of the machine learning classifiers does require two important distinctions, in case of dealing with categorical or numerical data. Whereas one-hot encoding is used for categorical features, numerical features are standardized by removing their mean and by scaling them to unit variance. To perform the standardization, the mean and the standard deviation values are computed from the training set of the initial center from a scaling function, which is used to standardize all data of both centers. After one-hot encoding, the clinical data shown in alphabetical order in Table A3 (Appendix B) resulted in 22 features.

It should be noted that not all classifiers are required for one-hot encoding or standardization. In fact, pre-processing was omitted for XGBoost, since it required only the replacement of categorical instances with numerical values, and also for CatBoost since it has internal and dedicated pre-processing for categorical data.

#### 2.2.4. Class-Balancing Strategies and Learning Approaches

The TAVI procedure has a high chance of success and only a small fraction of the patients have non-favorable outcomes. This results in an imbalanced class problem [28] which was addressed with three approaches:

(1) Balanced class weighting (as ClassWeight),
(2) Random oversampling of the minority class (as RandomOvers),
(3) SMOTE-NC for the minority class.

Including balanced class weighting [29] for each classifier training was the first solution that was adopted. Random oversampling of the minority class was applied prior to training each classifier until an equal proportion between classes was reached. Finally, the SMOTE-NC (synthetic minority oversampling technique for nominal and continuous) technique [30,31] was tested.

A total amount of six machine learning classifiers (I-VI) have been exploited to generate different prediction models and will be presented in the following order. (I) Support vector machine classifier (SVC) and (II) logistic regression (LR) represent the core of ML, since they are robust and widespread across multiple fields of research. (III) Random forest (RF), (IV) XGBoost (XGB) and (V) CatBoost (CatB) were included because they are based on decision trees, which are optimal for dealing with categorical features common in clinical data. Lastly, (VI) neural networks (NN) are considered for their recent exponential growth due to the widespread successful use in data and image analysis. An explanation for the classification algorithm of each classifier is provided in the Supplementary Materials.

### 2.3. Validation and Evaluation Metrics

Validation of the model has been performed with a stratified twentyfold cross-validation, so that 5% of the entire dataset was used as test set for internal validation per fold. From the remaining data (95%), 90% was used as training set and 10% as validation set. The validation set was employed to perform some work on the optimal hyperparameter setting to verify optimal convergence of the classifiers during the training process. Therefore, the validation set (in some cases within a tenfold cross-validation (CV) on the training set as specified in the section on hyperparameters of the Supplementary Materials) was used to find the optimal parameters for each model, to identify the best classifier on the validation score for each fold individually.

Despite being computationally intensive, this type of validation required three different split sets (training, validation and test set). This approach was chosen to avoid overoptimistic results, to reduce overfitting and to increase the generalization capabilities of the classifiers on another center population, guiding the training process to an optimal convergence.

Once each classifier was trained and tuned on the original center data, to generate the optimal prediction models for each fold, all models were transferred to the other center to perform the external validation. The pre-processing stage per fold was also transferred to guarantee common and identical data processing for the two different datasets.

The external validation was performed with a similar approach; thus, a stratified twentyfold CV on the other center data was used to assess the performance of each model. It is worth mentioning that each of the 20 models generated per classifier on the original center data were validated on one unique fold of the other center data. This approach was chosen to guarantee a similar validation method and metric to the one used for the internal validation.

The main purpose of this study was to assess the capability of several classifiers to discriminate across two groups and two different centers. Proper calibration of the models was not required, since the development of a risk score was beyond the scope of research. However, a calibration study was performed to provide insights related to the probability distributions obtained on the two center populations.

The area under the curve (AUC) of the receiver operating characteristic (ROC) (known as c-statistic) and Brier loss score were computed and reported for all classifiers, while calibration curves and ROC curves were reported for the top-three classifiers (based on AUC of the ROC) of each center and experiments, for both the internal and external validations results.

### 2.4. Experimental Setup

Two independent experiments have been performed, one considering CZE-TU/e as initial center (IC: CZE-TU/e) and AMC as validation center (VC: AMC), and vice versa. These two experiments were of crucial importance to perform the external validation with a bi-directional approach to both centers, in other words an inter-center cross-validation.

For each of the two experiments, two evaluations were required. At first, an internal evaluation of the models was performed to assess the prediction potential of the classifiers on the initial center data. Second, an external evaluation of the identical models was required to assess the prediction potential of the classifiers on the external center data. To provide reliable evaluation scores that could be later compared between both centers, a specific twentyfold cross-validation approach for both centers was developed. Both centers’ datasets were split into twenty stratified folds and these folds were used for both the internal and the external evaluations across both centers’ experiments. A total amount of twenty AUC values of the ROC were obtained to evaluate and assess the prediction potential for all the models. Final scores consisted of mean, standard deviation and interval of confidence of the twenty AUC values per classifier, sub-experiments (class-balancing) and experiments (cross-validation across centers). All comparisons between the internal and external validation results were based on these evaluation metrics.

All experiments were performed on Python 3.6.9 using scikit-learn library 0.21.3.

## 3. Results

**Figure 5:**
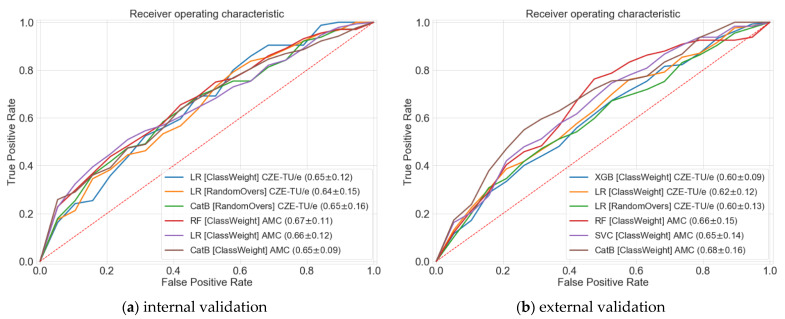
Area under curve (AUC) of the receiver operating characteristic (ROC) for the top-three classifiers per center with the highest validation score are shown with different colors. Both centers internal validation (**a**) and external validation (**b**) AUC metrics are shown jointly in this figure to highlight the similarity.

**Figure 6:**
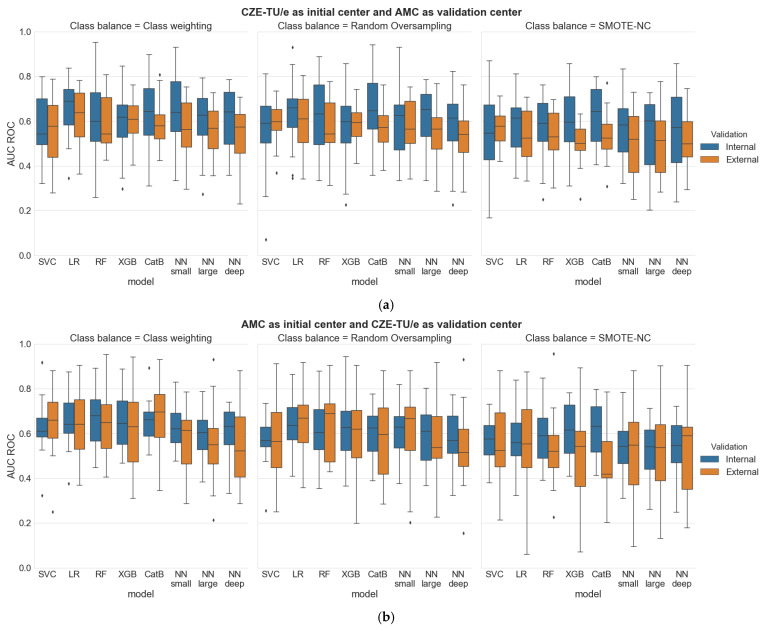
Boxplot results considering (**a**) CZE-TU/e as initial center and AMC as validation center and considering (**b**) AMC as initial center and CZE-TU/e as validation center. Both the internal and external validations per classifier are shown in blue and orange, respectively.

In this section the two different populations and all experimental results are presented in detail. At first, a comparison between the internal validation of the two centers is performed. Then, the internal and external validation results are compared, considering CZE-TU/e as initial center and AMC as validation center, and vice versa. All results are shown in Table A1 (Appendix A), and top-three results per center for both the internal and external validations are shown in Figure 5. Figure 6 portrays the distributions of all the results while Table 1 presents a final evaluation for both centers.

### 3.1. Analysis of the Data Distributions across the Centers

An analysis for comparison of the two different center populations was needed for assessing the main differences between the two populations, due to possible variations in the distribution of the clinical data. The distributions of the numerical and categorical features for both centers are displayed with boxplots and bars in Figure 4 and Figure A2 (Appendix B), respectively. Alternatively, category occurrences for each of the categorical features and missing value information for all clinical features can be obtained for further analysis from Table A3 (Appendix B).

No major differences were found between the two populations. However, some numerical and categorical features showed different distributions. The main differences can be explained by the different standard deviations across the two populations. In some cases, the quantity of statistical outliers is also different, but in most cases when outliers are present, they appear to occur for both centers jointly above or below the median value, therefore not resulting in an asymmetry between the two centers’ distributions. In some cases, the non-survived group showed a higher or lower median value with respect to the survived group; these corresponded across both populations. Exceptions are aortic valve mean gradient, body mass index, creatinine and hemoglobin. However, by including the outliers and by considering the mean values solely, these differences appear to reduce.

### 3.2. Internal and External Validations

The first part of the two experiments consisted in the internal validation of CZE-TU/e and AMC. The classifiers that showed the highest AUC results for CZE-TU/e were: Logistic regression (ClassWeight) with 0.65 ± 0.12, Logistic regression (RandomOvers) with 0.64 ± 0.15 and CatBoost (RandomOvers) with 0.65 ± 0.16. For the AMC, the highest AUC results were reached by: Random forest (ClassWeight) with 0.67 ± 0.11, Logistic regression (ClassWeight) with 0.66 ± 0.12 and CatBoost (ClassWeight) with 0.65 ± 0.09. AUC curves for the classifiers mentioned above can be found in Figure 5 (left). The highest results were obtained with balanced-class weighting and random oversampling. Therefore, only these two class-balancing strategies will be further discussed. However, all the AUC results for each class-balancing strategy can be found in Table A1 (Appendix A).

The second part of the two experiments consisted in the external validation for models trained on CZE-TU/e data and tested on AMC data, and vice versa. The classifiers that showed the highest external validation results for models trained on CZE-TU/e were: XGBoost (ClassWeight) with 0.60 ± 0.09, logistic regression (ClassWeight) with 0.62 ± 0.12 and logistic regression (RandomOvers) with 0.60 ± 0.13. For AMC, the highest external validation results for models trained on AMC data were reached by: Random forest (ClassWeight) with 0.66 ± 0.15, Support vector machine classifier (ClassWeight) with 0.65 ± 0.14 and CatBoost (ClassWeight) with 0.68 ± 0.16. AUC curves for the classifiers mentioned above can be found in Figure 5 (right).

A calibration study was performed for the top-three classifiers shown in Figure 5. The results of the study can be found in the Supplementary Materials with the Brier Score loss of each classifier involved in the experiments.

### 3.3. Final Evaluation

All distributions for all the results collected during the two experiments (both internal and external validations) that were performed are displayed in Figure 6.

From a first visual inspection of the results, it can be clearly noticed that all the distributions show a high standard deviation. Boxplots results show, as an overall impression, that when the models are generated by using the dataset of CZE-TU/e, lower results are observed in the external validation, but when the models are generated by using the dataset of AMC, more similar and sometimes higher external validation results are observed. These observations are reflected by both the median value and the interquartile range of the boxplots. The top-three results per center, shown in Figure 5, and the highest AUC results for both the internal and the external validations, are marked in Table 1, in bold font and between asterisks, respectively.

The first experiment with CZE-TU/e as initial center and AMC as validation center has shown that the best classifiers, based on AUC metrics, were logistic regression (ClassWeight) with 0.65 ± 0.12 for the internal validation and with 0.62 + 0.12 for the external validation, and logistic regression (RandomOvers) with 0.64 ± 0.15 for the internal validation and 0.60 ± 0.13 for the external validation. A performance drop at the external validation, ranging from 5–7%, is observed.

The second experiment with AMC as initial center and CZE-TU/e as validation center has shown that the best classifiers, based on AUC metrics, were random forest (ClassWeight) with 0.67 ± 0.11 for the internal validation and with 0.66 ± 0.15 for the external validation, and CatBoost (ClassWeight) with 0.65 ± 0.09 for the internal validation and 0.68 ± 0.16 for the external validation. A performance drop of 2% at the external validation is observed for random forest, while an improvement in the performance of 4% at the external validation is noticed for CatBoost.

## 4. Discussion

### 4.1. Feature Representation and Distributions

The initial setup of two distributions for each center is interesting for comparison but also invokes some issues. It was seen that missing values, missing features and different feature representations are obstacles which can interfere with the inter-center cross-validation purpose. In some cases, the amount of missing data was too high to allow data usage but in most of the cases data harmonization was possible. By imposing some decision rules (if missing data is above 80%, the feature is ignored, with lower numbers harmonization is typically possible but with a fair weighting of exclusion of specific cases to avoid penalization of centers). Different feature representations can be properly harmonized across the two centers to prepare the datasets for an optimal and fair cross-validation. The possibility to make a center-based comparison makes this effort of data harmonization worthwhile to perform.

Feature distributions for the two sub-groups of survived and non-survived to one-year patients should be taken into account before adopting any imputation techniques for missing values. The imputation can be a unique shared imputation or a center-dependent imputation, as in this study.

### 4.2. Dataset and Related Validation Aspects

The metrics that have been provided with this inter-center cross-validation approach are completely exempt of any information leak that could occur from the test set to the validation or training set. In fact, in both conducted experiments, both centers were blind relative to each other, leading to a strict validation approach which is often non-typical for other studies available in literature [14,15,21,22,23,37,38]. This makes our inter-center cross-validation very valuable and unique.

With respect to the TAVI procedure our work has identified the best classifiers for a center to model their data. These classifiers are then interesting candidates for validation at other centers. This makes the validation highly interesting for both centers. Furthermore, it can be noticed that a larger dataset can offer similar and sometimes higher prediction accuracy with respect to a smaller dataset.

Concerning the class-balancing strategies, random oversampling of the minority class has shown lower or similar results with respect to the class-balancing strategy for both the internal and external validations. On the other hand, SMOTE-NC shows poor prediction accuracy with respect to the other class-balancing strategies, possibly because the data interpolation is introducing too much variability and redundant information into the data.

### 4.3. Discussion on the Obtained Results

A high standard deviation in the AUC results is observed. This is mainly due to the relatively small sample size of the test set. Using a larger dataset or a reduced number of folds for the *k*-fold cross-validation would decrease the standard deviation, but most certainly also the validation results. This is because a lower number of patients would have been taken into account in the training set to generate the models.

When logistic regression is used on the smaller dataset of CZE-TU/e, it provides higher prediction accuracy with respect to the one obtained by all the other classifiers. However, when applying support vector machine, this leads to a low prediction accuracy when trained on the smaller dataset of CZE-TU/e. XGBoost, Random Forest and CatBoost have shown similar or better results with respect to logistic regression on the larger dataset of AMC. In general, neural networks have shown good prediction accuracy on the internal validations for both centers. However, poor results have been obtained for the external validations, exhibiting the highest drop in accuracy at the external validation.

The previous discussion on the prediction accuracy with various classifiers is confirmed by the presented boxplots results, which show on average, that when the models are generated using the dataset of CZE-TU/e, somewhat lower results are obtained for the external validation, while when the models are generated using the dataset of AMC, more similar or sometimes even higher results are achieved for the external validation. This phenomenon is related to the amount of available data for machine learning. Logistic regression (ClassWeight and RandomOvers) shows to be ideal for generating the models with the small-scale dataset, while random forest (ClassWeight) and CatBoost (ClassWeight) seem to be ideal for generating models with the large-scale dataset, proving to reach high-performance results. This can be explained by the fact that these classifiers have a tree-based decision structure which can handle categorical features and can capture non-linear patterns.

### 4.4. Future Work

Given the results discovered in this inter-center cross-validation study further developments of this research can be envisioned. A further logical step would be the generation of a unique model, by combining the two centers data for the development set. This would lead to the creation of an improved model incorporating a much larger amount of information. However, this achievement is severely limited by the data-sharing policies of the medical centers, preventing data combinations of multiple centers. To this end, future approaches for more general setups could be based on federated (or collaborative) learning or more specifically distributed (or split) learning for neural networks.

## 5. Limitation of the Study

The relatively small population size shared between the two centers poses some constraints on the generalization capabilities of the joint model for larger datasets. The window time for the data acquisition of the Amsterdam Medical Center is large [24] compared to lower amount of data available at top-clinical hospitals. However, this benefit cannot always be fully exploited because if the time window becomes large, developments in techniques change over time within the data capturing window, which makes the data exploitation less fitted to the actual problem and technology.

The proposed validation approach does not require data exchange but does imply a clear and ongoing communication, a good collaboration and a good synchronization between the two centers, which was fortunately possible in our case but can sometimes be complicated for other combinations of centers.

## 6. Conclusions

With this research, we aim to perform an inter-center cross-validation of several prediction models of one-year mortality for patients who undergo the TAVI procedure, by exploiting several machine learning classifiers. Despite the advancement of machine learning in healthcare, data-sharing regulations are very strict and they do not always allow for exchanging the patient data, without the involvement of specific ethical committees’ protocols for data-sharing approval. In this study, we performed two external validations for two TAVI centers, which have been achieved by exchanging the models rather than exchanging the patient data. Multiple and different mortality prediction models have been developed and optimized on the initial center data, with different populations of 1300 and 631 TAVI patients, based on employing various machine learning techniques. Assessing missing values and feature distributions is essential for both analyzing the main differences across the two populations, but also for a careful selection of the clinical features that can be used in the joint modeling. We have proven that the prediction accuracy of models generated at one TAVI center can be extended to another TAVI center. Moreover, training with a larger dataset can offer similar or higher prediction accuracy on the external validation with a smaller dataset, while training on a smaller dataset offers lower prediction accuracy on the external validation with a larger dataset. This research study is an extended cross-validation performed across two TAVI centers and it proposes a unique and scalable exchange protocol for validation without any data exchange. With respect to the TAVI procedure, the fact that our work has identified the best classifiers for a center to model their data and to test that model with the data of another center makes the experiments mutually highly interesting for both centers. The research can be further extended to larger datasets for the mortality prediction for TAVI procedure but also to any other clinical scenario where data sharing is not possible.

## Figures and Tables

**Table 1:** Final evaluation, internal and external validation results comparisons based on mean AUC of the ROC curve. AUC is shown as mean and the improvement or drop in the performance of the classifier (Δ) in expressed in percentage with respect to the mean AUC of the internal and of the external validation.

		Class-Balancing Strategy					
		Balanced-Class Weighting			Random Oversampling		
		Internal eval.	External eval.	Δ (%)	Internal eval.	External eval.	Δ (%)
SVC ^1^	IC: CZE-TU/e, VC: AMC	0.58	0.55	−5%	0.55	0.59	+7%
	IC: AMC, VC: CZE-TU/e	0.63	**0.65**	+3%	0.57	0.58	+2%
LR ^2^	IC: CZE-TU/e, VC: AMC	*0.65*	*0.62*	−5%	**0.64**	**0.60**	−7%
	IC: AMC, VC: CZE-TU/e	**0.66**	0.65	−2%	0.65	0.64	−2%
RF ^3^	IC: CZE-TU/e, VC: AMC	0.62	0.58	−7%	0.63	0.58	−9%
	IC: AMC, VC: CZE-TU/e	*0.67*	*0.66*	−2%	0.61	0.64	+5%
XGBoost ^4^	IC: CZE-TU/e, VC: AMC	0.60	**0.60**	0%	0.58	0.59	+2%
	IC: AMC, VC: CZE-TU/e	0.64	0.62	−3%	0.62	0.59	−5%
CatBoost ^5^	IC: CZE-TU/e, VC: AMC	0.63	0.59	−7%	**0.65**	0.58	−12%
	IC: AMC, VC: CZE-TU/e	*0.65*	*0.68*	+4%	0.61	0.58	−5%
NN small ^6^	IC: CZE-TU/e, VC: AMC	0.64	0.57	−12%	0.59	0.59	0%
	IC: AMC, VC: CZE-TU/e	0.63	0.57	−11%	0.61	0.60	−2%
NN large ^7^	IC: CZE-TU/e, VC: AMC	0.60	0.55	−9%	0.62	0.54	−15%
	IC: AMC, VC: CZE-TU/e	0.60	0.54	−11%	0.58	0.56	−4%
NN deep ^8^	IC: CZE-TU/e, VC: AMC	0.62	0.55	−13%	0.58	0.52	−12%
	IC: AMC, VC: CZE-TU/e	0.61	0.54	−13%	0.58	0.54	−7%

^1^ Support-vector machine classifier [32], ^2^ Logistic regression, ^3^ Random Forest [33], ^4^ Extreme gradient Boosting [34], ^5^ CatBoost [35,36], ^6^ Neural network small 12 neurons-2 layers, ^7^ Neural network large 140 neurons-2 layers, ^8^ Neural network deep 84 neurons-3 layers. Top-three results per center are marked in bold font. The highest AUC results, reported in the abstract, for both the internal and the external validations are marked between asterisks.

**Figure A1:**
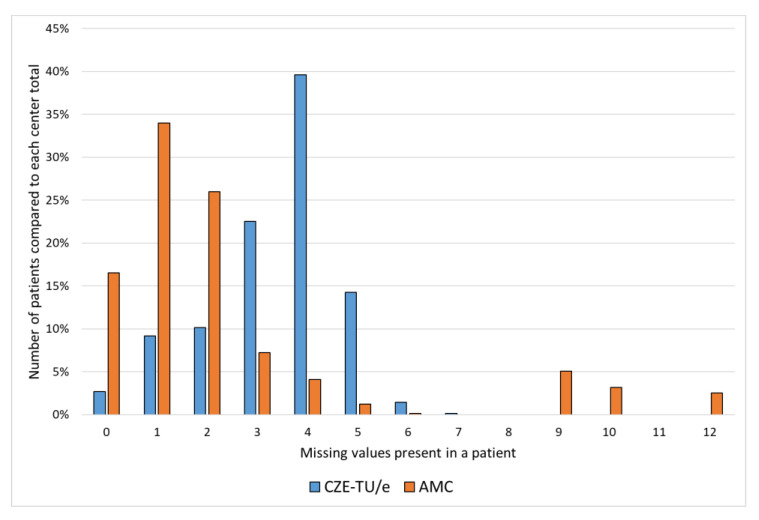
Distribution of missing values per patient on each center population.

## Data Availability

The data presented in this study are not publicly available due to privacy and ethical restrictions. Data for CZE-TU/e were obtained from Catharina Hospital (Eindhoven, the Netherlands) and have been made available for Eindhoven university of technology (Eindhoven, the Netherlands) after the permission and approval, through formal request, of the Catharina Hospital ethical committee. Data for AMC were collected in Amsterdam UMC (Amsterdam, the Netherlands). The models presented in this study are available for further validation and cross-validation studies with other centers. Contact the corresponding authors to this extent.

## Supplementary Materials

The TRIPOD statement is available online at https://www.mdpi.com/article/10.3390/jcdd8060065/s1. Details about the TAVI patient, each classifier with its hyperparameters and the calibration study conducted for the models are available online at https://www.mdpi.com/article/10.3390/jcdd8060065/s1.

## Appendix A

**Table A1:** Final evaluation, internal and external validation results comparisons based on AUC of the ROC curve. AUC is shown as mean ± standard deviation, within brackets are the confidence intervals at 95%.

		Class-Balancing Strategy								
		Balanced-Class Weighting			Random Oversampling			Smote-NC		
		Internal eval.	External eval.	Δ (%)	Internal eval.	External eval.	Δ (%)	Internal eval.	External eval.	Δ (%)
SVC ^1^	IC: CZE-TU/e VC: AMC	0.58 ± 0.14, [0.51, 0.65]	0.55 ± 0.16, [0.47, 0.62]	−5%	0.55 ± 0.17, [0.47, 0.63]	0.59 ± 0.09, [0.55, 0.64]	+7%	0.55 ± 0.18, [0.46, 0.63]	0.57 ± 0.08, [0.53, 0.60]	+4%
	IC: AMC VC: CZE-TU/e	0.63 ± 0.11, [0.57, 0.68]	0.65 ± 0.14, [0.59, 0.72]	+3%	0.57 ± 0.09, [0.53, 0.62]	0.58 ± 0.17, [0.50, 0.66]	+2%	0.56 ± 0.10, [0.51, 0.61]	0.55 ± 0.16, [0.47, 0.63]	−2%
LR ^2^	IC: CZE-TU/e VC: AMC	0.65 ± 0.12, [0.60, 0.71]	0.62 ± 0.12, [0.56, 0.68]	−5%	0.64 ± 0.15, [0.57, 0.72]	0.60 ± 0.13, [0.54, 0.66]	−7%	0.58 ± 0.12, [0.52, 0.64]	0.53 ± 0.12, [0.47, 0.58]	−9%
	IC: AMC VC: CZE-TU/e	0.66 ± 0.12, [0.60, 0.71]	0.65 ± 0.14, [0.58, 0.71]	−2%	0.65 ± 0.11, [0.59, 0.70]	0.64 ± 0.15, [0.57, 0.71]	−2%	0.57 ± 0.12, [0.52, 0.63]	0.55 ± 0.21, [0.45, 0.65]	−4%
RF ^3^	IC: CZE-TU/e VC: AMC	0.62 ± 0.16, [0.54, 0.70]	0.58 ± 0.11, [0.53, 0.64]	−7%	0.63 ± 0.16, [0.55, 0.70]	0.58 ± 0.12, [0.52, 0.63]	−9%	0.56 ± 0.14, [0.49, 0.63]	0.54 ± 0.11, [0.48, 0.59]	−4%
	IC: AMC VC: CZE-TU/e	0.67 ± 0.11, [0.61, 0.72]	0.66 ± 0.15, [0.59, 0.73]	−2%	0.61 ± 0.13, [0.55, 0.67]	0.64 ± 0.14, [0.57, 0.70]	+5%	0.59 ± 0.12, [0.53, 0.65]	0.53 ± 0.15, [0.46, 0.61]	−11%
XGBoost ^4^	IC: CZE-TU/e VC: AMC	0.60 ± 0.14, [0.53, 0.66]	0.60 ± 0.09, [0.56, 0.65]	0%	0.58 ± 0.18, [0.50, 0.67]	0.59 ± 0.08, [0.56, 0.63]	+2%	0.60 ± 0.14, [0.53, 0.67]	0.50 ± 0.09, [0.46, 0.55]	−20%
	IC: AMC VC: CZE-TU/e	0.64 ± 0.12, [0.59, 0.70]	0.62 ± 0.16, [0.54, 0.70]	−3%	0.62 ± 0.15, [0.55, 0.69]	0.59 ± 0.19, [0.50, 0.68]	−5%	0.62 ± 0.12, [0.56, 0.67]	0.50 ± 0.19, [0.41, 0.59]	−24%
CatBoost ^5^	IC: CZE-TU/e VC: AMC	0.63 ± 0.15, [0.56, 0.70]	0.59 ± 0.11, [0.54, 0.65]	−7%	0.65 ± 0.16, [0.57, 0.72]	0.58 ± 0.10, [0.53, 0.63]	−12%	0.62 ± 0.13, [0.56, 0.68]	0.53 ± 0.10, [0.48, 0.58]	−17%
	IC: AMC VC: CZE-TU/e	0.65 ± 0.09, [0.61, 0.70]	0.68 ± 0.16, [0.60, 0.75]	+4%	0.61 ± 0.11, [0.55, 0.66]	0.58 ± 0.17, [0.50, 0.66]	−5%	0.62 ± 0.11, [0.56, 0.67]	0.47 ± 0.15, [0.40, 0.55]	−32%
NN small ^6^	IC: CZE-TU/e VC: AMC	0.64 ± 0.16, [0.56, 0.72]	0.57 ± 0.13, [0.51, 0.63]	−12%	0.59 ± 0.16, [0.51, 0.67]	0.59 ± 0.11, [0.53, 0.64]	0%	0.58 ± 0.12, [0.52, 0.63]	0.50 ± 0.14, [0.43, 0.56]	−16%
	IC: AMC VC: CZE-TU/e	0.63 ± 0.10, [0.59, 0.68]	0.57 ± 0.14, [0.50, 0.63]	−11%	0.61 ± 0.11, [0.56, 0.66]	0.60 ± 0.18, [0.52, 0.69]	−2%	0.54 ± 0.12, [0.49, 0.60]	0.53 ± 0.21, [0.43, 0.63]	−2%
NN large ^7^	IC: CZE-TU/e VC: AMC	0.60 ± 0.14, [0.54, 0.67]	0.55 ± 0.11, [0.50, 0.61]	−9%	0.62 ± 0.13, [0.56, 0.68]	0.54 ± 0.13, [0.48, 0.60]	−15%	0.55 ± 0.15, [0.48, 0.62]	0.50 ± 0.14, [0.43, 0.56]	−10%
	IC: AMC VC: CZE-TU/e	0.60 ± 0.11, [0.55, 0.65]	0.54 ± 0.16, [0.47, 0.62]	−11%	0.58 ± 0.12, [0.52, 0.64]	0.56 ± 0.18, [0.47, 0.64]	−4%	0.53 ± 0.12, [0.47, 0.59]	0.53 ± 0.20, [0.43, 0.62]	0%
NN deep ^8^	IC: CZE-TU/e VC: AMC	0.62 ± 0.13, [0.55, 0.68]	0.55 ± 0.12, [0.49, 0.60]	−13%	0.58 ± 0.16, [0.50, 0.65]	0.52 ± 0.13, [0.46, 0.58]	−12%	0.56 ± 0.18, [0.48, 0.65]	0.51 ± 0.13, [0.45, 0.57]	−10%
	IC: AMC VC: CZE-TU/e	0.61 ± 0.11, [0.56, 0.66]	0.54 ± 0.16, [0.46, 0.62]	−13%	0.58 ± 0.12, [0.53, 0.64]	0.54 ± 0.16, [0.46, 0.61]	−7%	0.53 ± 0.14, [0.47, 0.60]	0.53 ± 0.21, [0.43, 0.63]	0%

^1^ Support-vector machine classifier, ^2^ Logistic regression, ^3^ Random Forest, ^4^ Extreme gradient Boosting, ^5^ CatBoost, ^6^ Neural network small 12 neurons-2 layers, ^7^ Neural network large 140 neurons-2 layers, ^8^ Neural network deep 84 neurons-3 layers.

## Appendix B

**Table A2:** Clinical data harmonization stage. Summarized feature detail and difference for the two centers: Amsterdam UMC (AMC) and Catharina hospital of Eindhoven (CZE-TU/e).

Feature Name	Data Type and Representation		Common Representation
	CZE-TU/e	AMC	
Aortic valve regurgitation	Categorical Instances: [No, mild, moderate, severe]	Categorical Instances: [No, mild, moderate, severe]	Not included due to the amount of missing values
Atrio ventricular block *	Categorical Instances: [No, AV-block I, AV-block II (Wenckebach), AV-block II (Mobitz), AV-block III]	Categorical Instances: [No, AV-block I, AV-block II, AV-block III]	Not included due to the amount of missing values
Chronic obstructive pulmonary disease	Categorical Instances: [No, GOLD class 1, GOLD class 2, GOLD class 3, GOLD class 4, GOLD class unknown]	Categorical Instances: [No, GOLD class 1, GOLD class 2, GOLD class 3, GOLD class 4, GOLD class unknown]	Included, Categorical Instances: [No, Yes]
Diabetes	Categorical Instances: [No, type 1, type 2 (diet), type2 (oral antidiabetics), type 2 (insulin), type 2 (no treatment)]	Categorical Instances: [No, type 1, type 2]	Included, Categorical Instances: [No, Yes]
General health score of the quality-of-life questionnaire RAND-36 *	Numerical range: (0–100)	Not available	Not included because was not cross-available
Glomerular filtration rate (estimated) [eGFR]	Numerical unit: mL/min/1.73 m^2^ (estimated with MDRD formula)	Numerical unit: mL/min/1.73 m^2^ (estimated with CKD-EPI formula)	Not included due to the amount of missing values
Hematocrit *	Numerical unit: %	Numerical unit: %	Not included due to the amount of missing values
Left ventricle function or qualitative ejection function	Categorical Instances: [normal (>50%), moderate (31–50%), poor (21–30%),very poor (<20%)]	Categorical Instances: [Mild, moderate, moderate-severe, severe]	Not included due to the amount of missing values
Left ventricle quantitative ejection fraction	Numerical unit: %	Numerical unit: %	Not included due to the amount of missing values
N-terminal fragment pro-brain natriuretic peptide [NT-pro-BNP]	Numerical unit: pico mol/L	Numerical unit: nano gram/L	Not included due to the amount of missing values
QRS interval	Numerical unit: 10^−3^ s	Numerical unit: 10^−2^ s	Included, Numerical Unit: 10^−3^ s

* Previous individual center studies [19,20] highlighted relevant clinical features for predicting one-year mortality. However, some features were not available in both centers, so that their inclusion in this study was not possible.

**Table A3:** Summarized patient characteristics from Amsterdam UMC (AMC) and Catharina hospital of Eindhoven (CZE-TU/e).

Clinical Name	Unit or Instances	AMC			CZE-TU/e		
		Survived (at One-Year) (1171)	Non-Survived (at One-Year) (129)	*p*-Value	Survived (at One-Year) (564)	Non-Survived (at One-Year) (67)	*p*-Value
Age (AGE)	[years]	81 ± 7	83 ± 7	0.117	81 ± 6	80 ± 6	0.784
		(1170)	(130)		(564)	(67)	
Aortic Valve Area (AVA)	[cm^2^]	0.8 ± 0.2	0.8 ± 0.2	0.457	0.7 ± 0.2	0.7 ± 0.2	0.104
		(949)	(111)		(148)	(18)	
Aortic Valve Mean Gradient (AVMEANGRAD)	[mmHg]	43 ± 16	44 ± 19	0.764	46 ± 16	39 ± 20	0.199
		(622)	(69)		(137)	(15)	
Aortic Valve Peak Gradient (AVPEAKGRAD)	[mmHg]	68 ± 23	64 ± 26	0.122	77 ± 24	71 ± 32	0.445
		(956)	(117)		(172)	(19)	
Beta Blockers class of medicine (BETABLOCK)	No	41% (481)	33% (42)	0.044	88% (498)	79% (53)	0.052
	Yes	56% (651)	67% (86)		12% (66)	21% (14)	
Body Mass Index (BMI)	[kg/m^2^]	28.0 ± 5	27.0 ± 6	0.088	27.1 ± 4.4	26.2 ± 4.5	0.155
		(1134)	(130)		(563)	(67)	
Chronic obstructive pulmonary disease (COPD)	No	65% (756)	51% (66)	<0.001	82% (464)	72% (48)	0.045
	Yes	24% (282)	43% (55)		17% (98)	28% (19)	
Creatinine (CREAT)	[μmol/L]	98 ± 40	120 ± 55	<0.001	108 ± 62	116 ± 46	0.186
		(1126)	(130)		(558)	(67)	
Diabetes (DIABETES)	No	61% (720)	61% (79)	0.373	75% (421)	70% (47)	0.502
	Yes	27% (313)	33% (42)		25% (142)	30% (20)	
Hemoglobin (HEMOGLOB)	[mmol/L]	7.8 ± 1.0	7.7 ± 1.2	0.289	7.9 ± 1.0	7.5 ± 0.9	0.005
		(1123)	(129)		(358)	(45)	
New York Heart Association Functional Classification (NYHA)	1	3% (32)	1% (1)	0.001	2% (9)	6% (4)	0.112
	2	20% (236)	16% (20)		10% (59)	7% (5)	
	3	43% (507)	67% (86)		33% (184)	36% (24)	
	4	7% (80)	17% (22)		9% (49)	13% (9)	
Previous Devices (pacemakers) (PREVDEVICE)	No	83% (970)	81% (104)	0.166	74% (417)	54% (36)	0.011
	Yes	9% (103)	13% (17)		9% (52)	18% (12)	
Previous Myocardial Infarction (PREVMYOINF)	No	73% (852)	71% (91)	0.091	69% (387)	54% (36)	0.168
	Yes	16% (187)	23% (30)		19% (105)	24% (16)	
QRS complex time (QRS)	[msec]	104 ± 26	107 ± 27	0.424	110 ± 29	121 ± 33	0.014
		(344)	(52)		(556)	(63)	
Sex (SEX)	Male	44% (514)	50% (65)	0.191	54% (303)	69% (46)	0.028
	Female	56% (657)	50% (64)		46% (261)	31% (21)	
Smoking (SMOKING)	No	42% (490)	46% (59)	0.675	70% (392)	76% (51)	0.532
	Former	41% (479)	38% (49)		7% (41)	6% (4)	
	Yes	9% (104)	10% (13)		23% (131)	18% (12)	

Mean ± SD are shown for numerical features while the percentage is shown for categorical features. Population size is shown between brackets.

Distribution of the patients’ missing values for the two centers population are shown in Figure A1.
