# Supplementary material for: Machine Learning for Predicting Mortality in Transcatheter Aortic Valve Implantation: An Inter-Center Cross Validation Study
Source: J Cardiovasc Dev Dis. 2021 Jun 4;8(6):65. doi: 10.3390/jcdd8060065 (PMC8227005; doi:10.3390/jcdd8060065)
Supplement: Supplementary file 1 [file jcdd-08-00065-s001.zip › supplementary/jcdd-1233331-supplementary.pdf]

Supplementary Materials

The TAVI Patient (Extension of the Introduction)

Candidates for TAVI are often frail patients with several comorbidities and with a complicated medical history (diabetes, chronic obstructive pulmonary disease, myocardial infarction, pacemaker, conduction disorders, obesity, smoking) [1]. However, it is still a matter of debate why certain patients do not benefit or only gain limited advantages from the procedure [2]. This limited gain may be attributed to certain complications that can occur during or after the procedure, or the absence of improvement in the symptoms, so called futility.

It is essential to identify patients who are likely to have improvements (in their symptoms and in their quality of life) after procedure, and thus benefit from TAVI, and those who do not. However, estimating improvements is a non-trivial task due to the small amount of information which is available nowadays, collected with walking tests or questionnaires for example. In the future this could be facilitated by taking advantage of wearable devices exploiting sophisticated sensors allowing real-time data acquisition before and after the procedure to perform objective evaluations of patients in their daily routines.

Considering the current limitations and difficulties in the estimation of patient improvements utilizing mortality as patient assessment is, at the current time, the most objective information which is available in the medical registry. Through the development of a decision-support tool to predict mortality, based on large amount of retrospective clinical data, a further step forward in the research can be achieved. Especially the exploitation of recent machine learning techniques enable the disclosure of important and specific (linear or non-linear) patterns in the clinical data, which would not have been found otherwise. Related to these techniques, we propose an exchange protocol of models instead of medical data to mutually evaluate the quality of prediction models which adds further robustness to the decision-making.

Learning Approaches and Classifiers

A total amount of six machine learning classifiers (I–VI) have been exploited to generate different prediction models and will be presented in the following order. (I) Support vector machine classifier (SVC) and (II) logistic regression (LR) represent the core of ML, since they are robust and widespread across multiple fields of research. (III) Random forest (RF), (IV) XGBoost (XGB) and (V) CatBoost (CatB) were included because they are based on decision trees, which are optimal for dealing with categorical features common in clinical data. Lastly, (VI) neural networks (NN) are considered for their recent exponential growth due to the widespread successful use in data and image analysis. Here below a short explanation of the classification algorithm of each classifier.

(I) Support-vector machine classifier (SVC) [3] is one of the most robust techniques that is available for classification, since many decades. SVC is a technique that iteratively generate hyperplanes in a multidimensional space to divide the classes until a maximum marginal hyperplane that best divides the training data is found. SVC uses a kernel trick to deal with non-linearity, by transforming the input space to a higher dimension.

(II) Logistic regression (LR) classifier is a widespread and well-known technique in the clinical field, and because of its simplicity and transparency it offers trustable results, which, in this case, serves as a solid background for comparison with other classifiers. LR is defined as an advanced statistical technique that can be included also in the machine learning techniques. It is a classification algorithm that transforms the classification output by using the logistic sigmoid function to return a probability value which can then be mapped to the different classes.

(III) Random Forest (RF) [4] is an ensemble learning technique for decision trees, based on random bootstrap aggregation. RF consists of a large number of uncorrelated individual decision trees that operate as an ensemble learning method. This method consists in averaging multiple shallow decision trees, trained on different parts of the same training set, with the goal of reducing the variance, at the expense of a small increase in the bias and some loss of interpretability but generally greatly boosting the performance.

(IV) Extreme gradient Boosting (XGBoost or XGB) [5] is a gradient boosting on decision trees (GBDT) classifier designed for speed and performance. GBDT algorithms are an ensemble technique where new decision trees models are iteratively added to correct the residuals or errors of the model at the preceding iteration until a convergence is reached. A gradient descent algorithm is used to minimize the loss of the added models between successive iterations.

(V) CatBoost [6,7] is a GBDT classifier specifically designed for categorical features. A dedicated pre-processing converts all the categorical features into numerical data by incorporating the recurrence of each instance. Numerical features are then processed by aggregating different feature values in a histogram, which is specifically optimized for an efficient and fast memory access and elaboration. CatBoost then builds an ensemble model, in an iterative fashion, of decision trees to gradually reduce the training error.

(VI) Neural networks classifier (NN) is a technique that has shown a very high flexibility in the development of multiple architectures which are ready-to-be-used. A multilayer perceptron (MLP) is a neural network which consist of at least three fully-connected layers of nodes: an input layer, one or more hidden layers and an output layer. Each layer except the input uses a nonlinear activation function to map the weighted inputs to the output of each neuron. MLP utilizes a supervised learning technique for training, called backpropagation, which changes the connection weights after each subset of the dataset is processed. With a gradient approach, which is based on the amount of error computed in the output compared to the expected result, the connection weights are updated within successive iterations.

Hyperparameter Optimization

A hyperparameter optimization was performed. A tenfold cross-validation on the training set was used to assess the best parameters for all the classifiers. Similar optimization was performed for the neural network architectures, however, by empirically exploring the amount of multiple architectures, layers and neurons. Three neural networks architectures are reported in the results section. They are identified as small, large and deep neural networks, depending on the number of neurons and fully-connected layers that are used: 12 neurons (2 layers) for small networks, 140 neurons (2 layers) for large networks and 84 neurons (3 layers) for deep networks.

Besides this optimization, 10% of the training set was used as validation set for XGBoost, CatBoost and neural network to optimize convergence of the training. The validation set was used to interrupt the learning process of the models by stopping the training of each model when the iteration count exceeded the optimal amount. A loss function was used as metric for measuring, at each iteration, the model convergence and by monitoring the loss curves, identifying the optimal iterations count to provide the optimal model. For support vector machines, logistic regression and random forest, it was not possible to monitor the learning process and the validation set was merged with the training data to enrich the training set. All iterated parameters for each classifier are shown in Table S1**Error! Reference source not found.**.

Table S1. Hyperparameter setting.

Classifier	Parameter	Iterated Values
SVC [1]	Max number of iterations	5000
	Kernel	Radial Basis Function, linear, polynomial (degree 3, 4, 5), sigmoid
	Gamma (not applicable for linear kernel)	0.001, 0.01, 0.1, 1
	Regularization parameter C	0.1, 1, 10, 100, 1000
LR [2]	Solver	Limited-memory Broyden–Fletcher–Goldfarb–Shanno (LBFGS)
	Maximum number of iterations to converge	1,000,000
RF [3]	Number of trees (iterations)	20, 50, 100, 200
	Tree depth	2, 3, 4, 8

	Minimum number of data points placed in a node before its split	2, 4, 6
	Minimum number of data points allowed in a leaf node	1, 2, 4
	Maximum number of features considered for splitting a node	auto
XGBoost [4]	Number of trees (iterations)	20, 50, 100, 200
	Tree depth	2, 3, 4, 8
	Learning rate	0.3, 0.1, 0.01, 0.001
	Minimum sum of instance weight (hessian) needed in a child	5, 10
	Minimum loss reduction required to make a split on a leaf node	0, 0.5, 1, 3
	Subsample ratio of the training instances	0.7, 1.0
	Subsample ratio of columns when constructing each tree	0.7, 1.0
CatBoost [5]	Number of trees (iterations)	20, 50, 100, 200
	Tree depth	2, 3, 4, 8
	Learning rate	0.05, 0.10, 0.15
	L2 leaf regularization term	3, 10
NN [6]	Architectures: Fully connected layers, neurons per layer, total number of neurons	2 layers, [Inp.-8-4-Outp.], 12 neurons<br>2 layers, [Inp.-100-40-Outp.], 140 neurons<br>3 layers, [Inp.-64-16-4-Outp.], 84 neurons
	Dropout (between all fully connected layers)	0.5
	Optimizer	Adam
	Learning rate	0.001
	Kernel and Output regularization penalty (and factor)	L2 (0.001)
	Activation function (and factor)	LeakyReLU (0.01)
	Training epochs (and batch size)	1500 (256 patients)

[1] Support-vector machine classifier, [2] Logistic regression, [3] Random Forest, [4] Extreme gradient Boosting, [5] CatBoost, [6] Neural network.

Calibration Study Analysis

A calibration study was performed considering, for each experiments and validation, all classifiers with their respective class balancing strategies. All results of the Brier Score loss can be found in Table S2**Error! Reference source not found.**.

Table S2. Comparison of the internal and external validation results based on Brier score loss. Brier loss is shown as mean ± standard deviation, within brackets are the confidence intervals at 95%.

		Class-Balancing Strategy					
		Balanced-Class Weighting		*Random Oversampling*		*Smote-NC*	
		Internal eval.	External eval.	Internal eval.	External eval.	Internal eval.	External eval.
SVC [1]	IC: CZE-TU/e<br>VC: AMC	0.10 ± 0.01,<br>[0.09, 0.10]	0.09 ± 0.01,<br>[0.09, 0.09]	0.11 ± 0.02,<br>[0.10, 0.12]	0.10 ± 0.01,<br>[0.09, 0.10]	0.12 ± 0.03,<br>[0.11, 0.14]	0.09 ± 0.00,<br>[0.09, 0.09]
	IC: AMC<br>VC: CZE-TU/e	0.09 ± 0.01,<br>[0.08, 0.09]	0.09 ± 0.01,<br>[0.09, 0.10]	0.10 ± 0.01,<br>[0.10, 0.10]	0.10 ± 0.01,<br>[0.10, 0.11]	0.11 ± 0.01,<br>[0.10, 0.11]	0.12 ± 0.02,<br>[0.11, 0.13]
LR [2]	IC: CZE-TU/e<br>VC: AMC	0.16 ± 0.07,<br>[0.13, 0.19]	0.16 ± 0.07,<br>[0.13, 0.19]	0.21 ± 0.04,<br>[0.19, 0.23]	0.22 ± 0.03,<br>[0.21, 0.23]	0.21 ± 0.04,<br>[0.19, 0.22]	0.14 ± 0.02,<br>[0.13, 0.15]
	IC: AMC<br>VC: CZE-TU/e	0.10 ± 0.04,<br>[0.08, 0.12]	0.11 ± 0.03,<br>[0.09, 0.12]	0.21 ± 0.02,<br>[0.20, 0.23]	0.20 ± 0.02,<br>[0.19, 0.21]	0.21 ± 0.02,<br>[0.20, 0.23]	0.20 ± 0.04,<br>[0.18, 0.22]
RF [3]	IC: CZE-TU/e<br>VC: AMC	0.14 ± 0.03,<br>[0.13, 0.16]	0.13 ± 0.03,<br>[0.11, 0.14]	0.13 ± 0.02,<br>[0.12, 0.14]	0.11 ± 0.01,<br>[0.10, 0.12]	0.15 ± 0.02,<br>[0.14, 0.16]	0.13 ± 0.01,<br>[0.12, 0.14]
	IC: AMC<br>VC: CZE-TU/e	0.21 ± 0.01,<br>[0.21, 0.22]	0.21 ± 0.01,<br>[0.20, 0.22]	0.13 ± 0.01,<br>[0.12, 0.14]	0.12 ± 0.01,<br>[0.11, 0.12]	0.15 ± 0.02,<br>[0.14, 0.16]	0.18 ± 0.02,<br>[0.17, 0.19]
XGBoost [4]	IC: CZE-TU/e<br>VC: AMC	0.20 ± 0.04,<br>[0.17, 0.22]	0.20 ± 0.03,<br>[0.19, 0.22]	0.13 ± 0.03,<br>[0.11, 0.14]	0.12 ± 0.02,<br>[0.11, 0.13]	0.14 ± 0.03,<br>[0.12, 0.15]	0.14 ± 0.02,<br>[0.13, 0.15]
	IC: AMC<br>VC: CZE-TU/e	0.18 ± 0.04,<br>[0.16, 0.20]	0.15 ± 0.06,<br>[0.13, 0.18]	0.11 ± 0.02,<br>[0.10, 0.12]	0.10 ± 0.02,<br>[0.09, 0.11]	0.12 ± 0.02,<br>[0.11, 0.13]	0.18 ± 0.03,<br>[0.16, 0.19]
CatBoost [5]	IC: CZE-TU/e<br>VC: AMC	0.19 ± 0.03,<br>[0.17, 0.20]	0.18 ± 0.03,<br>[0.17, 0.20]	0.11 ± 0.02,<br>[0.10, 0.12]	0.10 ± 0.01,<br>[0.10, 0.11]	0.13 ± 0.03,<br>[0.12, 0.15]	0.12 ± 0.02,<br>[0.12, 0.13]
	IC: AMC	0.21 ± 0.02,	0.20 ± 0.03,	0.10 ± 0.01,	0.10 ± 0.02,	0.12 ± 0.02,	0.18 ± 0.03,

	VC: CZE-TU/e	[0.20, 0.22]	[0.19, 0.22]	[0.09, 0.11]	[0.09, 0.11]	[0.11, 0.13]	[0.16, 0.19]
NN small [6]	IC: CZE-TU/e VC: AMC	0.19 ± 0.02, [0.18, 0.19]	0.19 ± 0.02, [0.18, 0.20]	0.16 ± 0.02, [0.15, 0.17]	0.15 ± 0.02, [0.14, 0.16]	0.16 ± 0.02, [0.16, 0.17]	0.13 ± 0.01, [0.12, 0.13]
	IC: AMC VC: CZE-TU/e	0.19 ± 0.01, [0.18, 0.19]	0.18 ± 0.01, [0.18, 0.19]	0.18 ± 0.01, [0.17, 0.19]	0.18 ± 0.01, [0.17, 0.18]	0.17 ± 0.01, [0.17, 0.18]	0.17 ± 0.02, [0.17, 0.18]
NN large [7]	IC: CZE-TU/e VC: AMC	0.14 ± 0.03, [0.12, 0.15]	0.14 ± 0.02, [0.13, 0.15]	0.12 ± 0.02, [0.11, 0.14]	0.12 ± 0.01, [0.11, 0.13]	0.14 ± 0.02, [0.13, 0.15]	0.11 ± 0.01, [0.11, 0.12]
	IC: AMC VC: CZE-TU/e	0.15 ± 0.03, [0.14, 0.16]	0.14 ± 0.03, [0.13, 0.15]	0.14 ± 0.02, [0.13, 0.15]	0.14 ± 0.01, [0.13, 0.14]	0.14 ± 0.02, [0.13, 0.15]	0.13 ± 0.02, [0.12, 0.14]
NN deep [8]	IC: CZE-TU/e VC: AMC	0.15 ± 0.03, [0.14, 0.17]	0.15 ± 0.02, [0.14, 0.16]	0.12 ± 0.02, [0.11, 0.13]	0.11 ± 0.01, [0.11, 0.12]	0.14 ± 0.02, [0.13, 0.15]	0.11 ± 0.01, [0.11, 0.12]
	IC: AMC VC: CZE-TU/e	0.16 ± 0.02, [0.15, 0.17]	0.15 ± 0.02, [0.14, 0.16]	0.14 ± 0.03, [0.13, 0.16]	0.14 ± 0.03, [0.13, 0.16]	0.15 ± 0.02, [0.14, 0.16]	0.14 ± 0.02, [0.13, 0.15]

[1] Support-vector machine classifier, [2] Logistic regression, [3] Random Forest, [4] Extreme gradient Boosting, [5] CatBoost, [6] Neural network small 12 neurons–2 layers, [7] Neural network large 140 neurons–2 layers, [8] Neural network deep 84 neurons–3 layers.

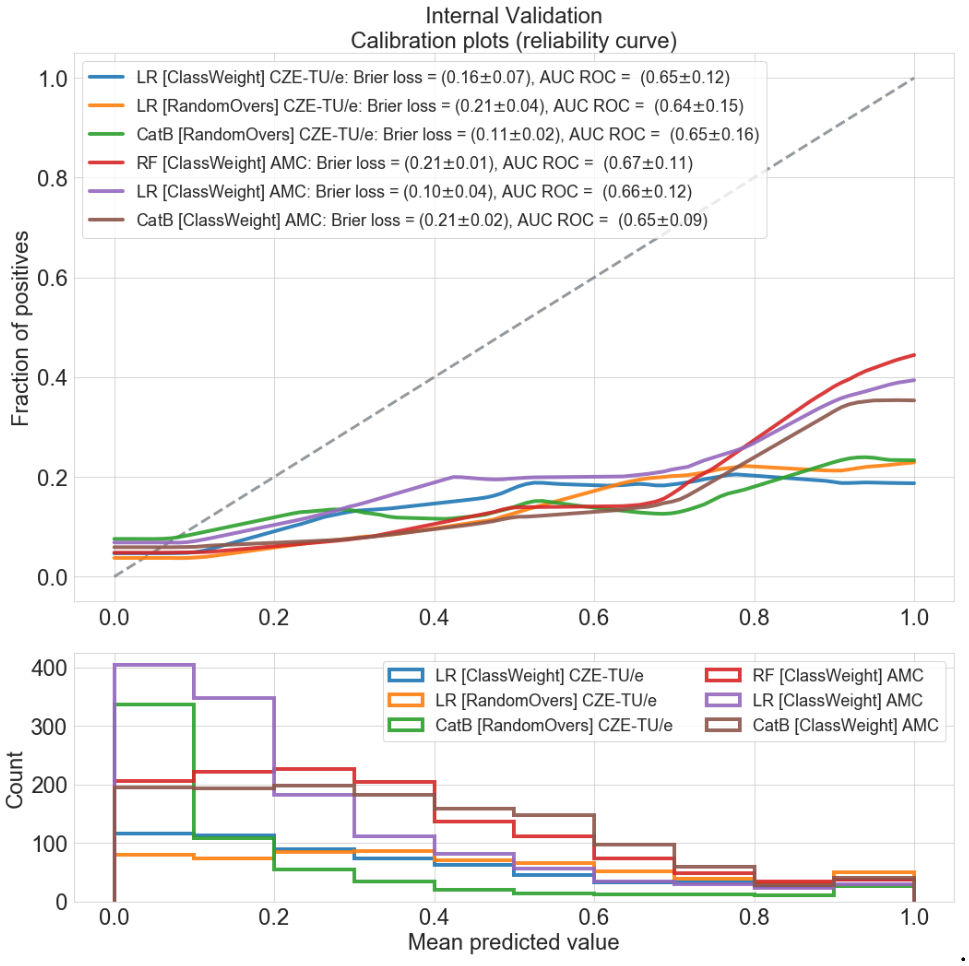

Figure S1. Calibration plots of the internal validation for the top-three classifiers per center. Both centers calibration plots the internal validation are shown jointly in this figure to highlight the similarity.

As shown in **Figure S1.** Calibration plots of the internal validation for the top-three classifiers per center.Figure S1, for the internal validation it can be clearly noticed that 20% and 25–40% of the predicted values of the non-survived group are within the range [0.8, 1.0] for CZE-TU/e and AMC, respectively. Furthermore 5–10% of the predicted values of the non-survived group are within the range [0.0, 0.1], hence representing false negative predictions.

All the other predicted values are reasonably distributed across the entire range [0.0, 1.0] for both centers. This can be clearly noticed by the nearly linear trend of the curves.

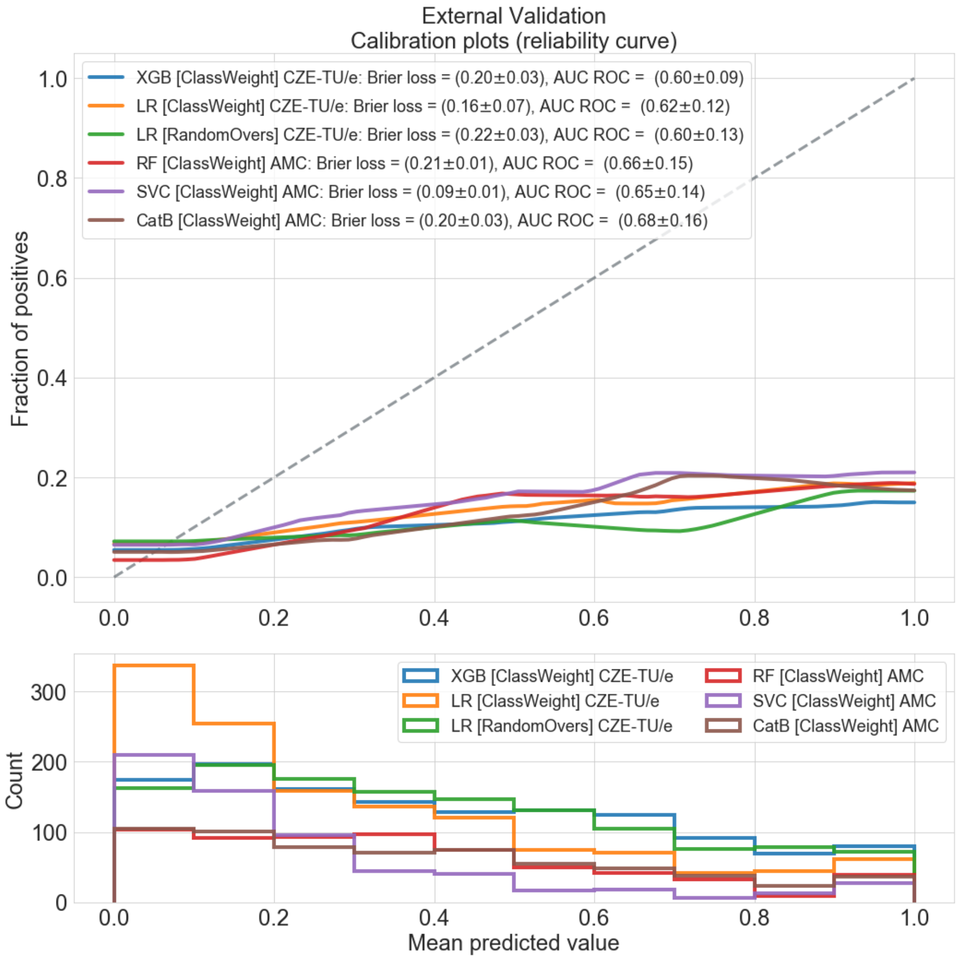

Figure S2. Calibration plots of the external validation for the top-three classifiers per center. Both centers calibration plots of the external validation are shown jointly in this figure to highlight the similarity.

As shown in Figure S2, similar distributions can be observed in the calibration plots of the external validation. With respect to the calibration plots of the internal validation, here only 20% of the predicted values of the non-survived group are within the range [0.8, 1.0], in this case for both centers. Similarly, to the calibration study of the internal validation, 5–10% of the predicted values of the non-survived group are within the range [0.0, 0.1].

All the predicted values are distributed over the whole range [0.0, 1.0] with a more evenly distribution than it was at the internal validation. However, the linear trend of the curves here shows a lower slope.

No noticeable differences are shown with respect to the calibration curves of the two centers. Some classifiers show a tendency to provide less extreme (ranges [0.0, 0.2] and [0.8, 1.0]) predicted values than other classifiers. However, there is no sufficient evidence to affirm that this is due to a specific classifier or to a specific class-balancing strategy.

To conclude, the calibration study showed uniform trends, for each of the models shown in the plot. This prove to facilitate a future calibration of the model, since the predicted values are mostly uniform on the entire interval. The linear trend of the curves shows a lower slope at the external validation, with respect to the internal validation. In other words, the predicted values of the non-survived group are sparser (or more scattered) across the entire range [0.0–1.0], than it was at the internal validation. This

observation is probably due to the differences in the data distribution across the two populations, which lead to more uncertain predictions on the other center population.

References

1. Baumgartner, H.; Falk, V.; Bax, J.J.; De Bonis, M.; Hamm, C.; Holm, P.J.; Iung, B.; Lancellotti, P.; Lansac, E.; Rodriguez Muñoz, D.; et al. 2017 ESC/EACTS Guidelines for the management of valvular heart disease. *Eur. Heart J.* **2017**, *38*, 2739–2791.
2. Puri, R.; Iung, B.; Cohen, D.J.; Rodes-Cabau, J. TAVI or No TAVI: Identifying patients unlikely to benefit from transcatheter aortic valve implantation. *Eur. Heart J.* **2016**, *37*, 2217–2225.
3. Cortes, C.; Vapnik, V. Support-vector networks. *Mach. Learn.* **1995**, *20*, 273–297.
4. Liaw, A.; Wiener, M. Classification and regression by randomForest. *R news* **2002**, *2*, 18–22.
5. Chen, T.; Guestrin, C. Xgboost: A scalable tree boosting system. In Proceedings of the Proceedings of the 22nd acm sigkdd international conference on knowledge discovery and data mining; ACM: San Francisco, CA, USA, 2016; pp. 785–794.
6. Prokhorenkova, L.; Gusev, G.; Vorobev, A.; Dorogush, A.V.; Gulin, A. CatBoost: Unbiased boosting with categorical features. In Proceedings of the Advances in Neural Information Processing Systems; Montréal, ON, Canada 2018; pp. 6639–6649.
7. Dorogush, A.V.; Ershov, V.; Gulin, A. CatBoost: Gradient boosting with categorical features support. *arXiv Prepr*. **2018**, arXiv1810.11363 2018.
